# Combination of T-DM1 and platinum-based chemotherapy in patient-derived xenograft models of muscle-invasive bladder cancer

**DOI:** 10.22038/IJBMS.2022.63509.14005

**Published:** 2022-07

**Authors:** Abolfazl Razzaghdoust, Samad Muhammadnejad, Mahmoud Parvin, Bahram Bahram, Masoumeh Zangeneh, Abbas Basiri

**Affiliations:** 1 Urology and Nephrology Research Center, Shahid Beheshti University of Medical Sciences, Tehran, Iran; 2 Gene Therapy Research Center, Digestive Diseases Research Institute, Tehran University of Medical Sciences, Tehran, Iran; 3 Department of Pathology, Labbafinejad Hospital, Shahid Beheshti University of Medical Sciences, Tehran, Iran; 4 Department of Oncology, Shohada-e-Tajrish Medical Center, School of Medicine, Shahid Beheshti University of Medical Sciences, Tehran, Iran; 5 Taleghani Hospital, Shahid Beheshti University of Medical Sciences, Tehran, Iran

**Keywords:** Bladder cancer, Chemotherapy, Patient-derived xenograft, Targeted therapy, T-DM1

## Abstract

**Objective(s):**

To assess the efficacy and safety of T-DM1, as an anti-HER2 antibody-drug conjugate (ADC), alone and in combination with two platinum-based chemotherapy regimens in patient-derived xenografts (PDXs) of muscle-invasive bladder cancer (MIBC) established in immunodeficient mice.

**Materials and Methods:**

After treatment initiation, tumor size was measured twice a week. Percent of tumor growth inhibition (TGI) and tumor response rates were calculated as efficacy endpoints. To evaluate treatment toxicity, relative body weight (RBW) was calculated for each group. For comparison of TGIs between treatment groups, the Kruskal-Wallis test was used. Also, the significance of the overall response (OR) rate between placebo groups with treatment groups was analyzed using Fisher’s exact test. Immunohistochemistry and fluorescence *in situ* hybridization techniques were used to evaluate the level of HER2 expression.

**Results:**

Our data showed that T-DM1 alone induced a moderate antitumor activity. While chemotherapy regimens induced a slight TGI when administered alone, interestingly, they showed strong antitumor activity when administered combined with T-DM1. The OR rates were higher when T-DM1 was combined with chemotherapy regimens than T-DM1 alone. When compared with the placebo group, the OR rates of combination groups were statistically significant. Our data also showed that the administered dose of each drug was well tolerated in mice.

**Conclusion:**

The combination of T-DM1 and platinum-based chemotherapy may represent a new treatment option for bladder tumors with even low HER2 expression, and could also provide substantial novel insight into tackling the challenges of MIBC management.

## Introduction

Muscle-invasive bladder cancer (MIBC) is one of the leading causes of mortality in genitourinary cancer patients (1). Platinum-based chemotherapy remains the mainstay of treatment in both the neoadjuvant and metastatic settings, but only less than half of patients experience a considerable tumor response to systemic chemotherapy (2). This emphasizes the need for developing more efficient treatments for MIBC.

Antibody-drug conjugates (ADCs) are considered a promising and fast-growing targeted anticancer therapy (3). Recently, ADC therapy for bladder cancer has attracted substantial interest (4). In contrast to systemic chemotherapy, ADCs more selectively target the tumor cells. Thus, in general, these targeted therapies are expected to enhance treatment efficacy and decrease side effects. Although ADCs targeting HER2 might be considered a promising targeted therapy for bladder cancer (5), targeted therapeutics directed at individual targets might be insufficient against tumor heterogeneity of bladder cancer (6). 

A strong rationale exists for combining ADCs and cytotoxic chemotherapy since chemotherapy drugs could increase tumor expression of ADC-catabolizing proteases (7). The combination of ADC therapy with systemic chemotherapy could open a promising era in the treatment of patients with MIBC. In this light, we hypothesized that T-DM1, as targeted therapy, in combination with systemic chemotherapy of MIBC could lead to improved tumor response in patient-derived xenografts (PDXs) established in immunodeficient mice. Therefore, the aim of this *in vivo* study was to assess the safety and efficacy of T-DM1 combined with platinum-based chemotherapy in urothelial cancer PDX models. This study may open new avenues for combination targeted therapy in MIBC patients and could provide major translational information for further investigation in the clinical situation. 

## Materials and Methods

***Patient-derived xenografts *** 

To investigate the combination efficacy of T-DM1 with platinum-based chemotherapy of bladder cancer, PDXs using female NOD.Cg- Prkdcscid Il2rgtm1Sug/ JicTac (NOG) mice were initially developed (8). Mice were provided by Omid Institute for Advanced Biomodels and kept in the animal lab of Digestive Diseases Research Institute, Tehran University of Medical Sciences (Tehran, Iran), in an individually ventilated cage system under specific pathogen-free conditions. All *in vivo* studies were carried out in accordance with guidelines outlined by the Institutional Ethics Committee, Declaration of Helsinki, and arrive guidelines for *in vivo* animal experiments** (9)**. The study was approved by the ethics committee of the Urology and Nephrology Research Center, Shahid Beheshti University of Medical Sciences (IR.SBMU.UNRC.1397.1). The PDXs were generated using fresh primary tumors from bladder cancer patients as previously described (8). Briefly, freshly resected patient tumors were sliced and engrafted subcutaneously on the flanks of NOG mice, under balanced anesthesia with 100 mg/kg ketamine and 10 mg/kg xylazine (Alfasan Co, Netherlands). Tumor growth was monitored by a caliper twice a week to establish the first passage of a PDX model. When the tumor size reached at least 1500 mm^3^, the tumor was passaged for expansion in later serial generations. Finally, PDX models from two patients were successfully developed (P8X20 and P8X26) and used for evaluation of drug efficacy.  The drugs were injected 68 days after tumor induction. Also, the volume of tumors (mean ± SEM) at the time of injection was 376.65 ± 52.32 mm^3^.

***Sample size and treatment groups***

For *in vivo* therapeutic studies, the number of mice required per group was determined by the “resource equation” method (10). Considering E ≥20 in the resource equation, and compensating for the expected attrition of two mice in each group, a total of seven mice per group was considered an adequate sample size for this study. A simple randomization method was performed by using the Excel 2016 software (Microsoft, USA), and 44 tumor-bearing NOG mice were randomly assigned to the following groups in a blinded fashion: Placebo; T-DM1; Gemcitabine/Cisplatin (Gem/Cis); Gemcitabine/Carboplatin (Gem/Carbo); T-DM1 + Gem/Cis; and T-DM1 + Gem/Carbo. 

***Determination of maximum tolerated dose*** 

A pilot study was conducted to determine a maximum tolerated dose (MTD) for each drug. Interspecies allometric scaling was used for conversion of human doses to mouse equivalent doses (MED) (11). At the initial dose levels, if any mice met the primary endpoint of >20% weight loss, the doses were considered to be toxic, and later doses were reduced incrementally to 50% of the former doses.  The following dose levels were considered safe and were set as the MTD for animal study:  3 mg/kg Cisplatin (EBEWE Pharma, Austria), 10 mg/kg Carboplatin (EBEWE Pharma, Austria), 40 mg/kg Gemcitabine (Sobhan Oncology, Iran), and 44 mg/kg T-DM1 (Roche, Switzerland) which was the initial dose level used without any sign of toxicity. A single intraperitoneal injection was performed for all groups. Also, intraperitoneal administration of 0.9% sodium chloride was done for the control group.

***Efficacy endpoints*** 

After treatment initiation, tumor size was blindly measured twice a week during a 6-week follow-up using a digital caliper, and the tumor volume was calculated as 0.5*length*width^2^. Growth curves were obtained by plotting the mean values of Relative tumor volume (RTV) on the y-axis against time on the x-axis, expressed as days after the start of treatment. RTV was defined as the mean tumor volume ratio between a given day and the day of treatment initiation. As a continuous efficacy endpoint, the percent of tumor growth inhibition (TGI), which is commonly used to assess therapeutic efficacy in PDXs, was calculated at study completion by using the following formula: %TGI = 100−(RTV treated/RTV control)*100. Significant growth inhibition was defined as a TGI of at least 50% (12). Furthermore, as a categorical efficacy endpoint, tumor response rates were calculated from changes in tumor volume over the course of treatment, based on RECIST criteria (13). As stated in previous studies (14), partial response (PR) was defined as RTVs ≤0.7 for two consecutive measurements over a seven-day period. Also, no evidence of any palpable tumor for two consecutive measurements over a seven-day period was classified as complete response (CR).  Overall response (OR) was defined as CR+PR. Mice reporting an RTV>1.2 were considered to have progressive disease (PD), and mice with neither sufficient shrinkage nor sufficient tumor volume increases were considered to have stable disease (SD).

***Toxicity endpoint***

To evaluate the treatment toxicity in each group, the weights of individual mice were measured twice a week. The body weight was presented as relative body weight (RBW). The RBW (%) on day *n* was calculated according to the following formula: RBW (%)=BW_n_/BW_0_*100, where BW_n_ is the body weight on day *n* and BW_0_ is the body weight on day 0.  A treatment was considered toxic in case of a mean weight loss >20% of the initial weight.  

***Immunohistochemistry*** 

At the end of the treatment, tumors were collected, weighed, and processed for formalin fixation. To evaluate the level of HER2 expression in patient samples and PDXs, standard immunohistochemistry (IHC) protocol was followed as previously described (8, 15, 16). Briefly, tissue slices were deparaffinized at 55 ^°^C for 10 min, cleared in xylene, and were then rehydrated by incubating in solutions with decreasing alcohol content. Then, antigen retrieval was done using Tris-EDTA buffer (pH 9.0) in a standard microwave for 34 min. The endogenous peroxidase was quenched with 3% H2O2 for 10 min. HER2 primary antibody (MAD-000308QD, Master Diagnostica, Spain) was selected to be used for overnight incubation at 4 ^°^C. Then, the sections were incubated with appropriate secondary antibodies (Detection kit; MAD-000237QK, Master Diagnostica, Spain) for 45 min. The slides were visualized with chromogen for 10 min. The sections were counterstained with hematoxylin, dehydrated in alcohol, cleared with xylene, and mounted for examination.

***Fluorescence in situ hybridization*** 

HER2 gene copy number was determined by using the fluorescence *in situ* hybridization (FISH) as described earlier (8). Briefly, formalin-fixed paraffin-embedded (FFPE) tumors were used to prepare sections, deparaffinized by xylene followed by rehydration.  Then, sections were immersed in a pepsin solution at 37 ^°^C, dehydrated, and allowed to dry. ZytoLight SPEC HER2/CEN 17 Dual Color Probe (Z-2020-20, ZytoVision GmbH, Germany) was used for hybridizations. Post-hybridization and detection processes were also performed according to the supplier’s protocol. 

***Statistical analyses*** 

All quantitative data are represented as mean ± SEM (standard error of the mean). Multigroup TGI comparisons were made using a Kruskal–Wallis test, followed by the Bonferroni adjustment. The significance of the OR rate between the placebo group with treatment groups was analyzed using Fisher’s exact test. A *P*-value<0.05 was considered statistically significant. The statistical analyses were performed using IBM SPSS, version 23 (IBM Corp., Armonk, NY, USA). 

## Results

***HER2 expression***  

**Figure 1:**
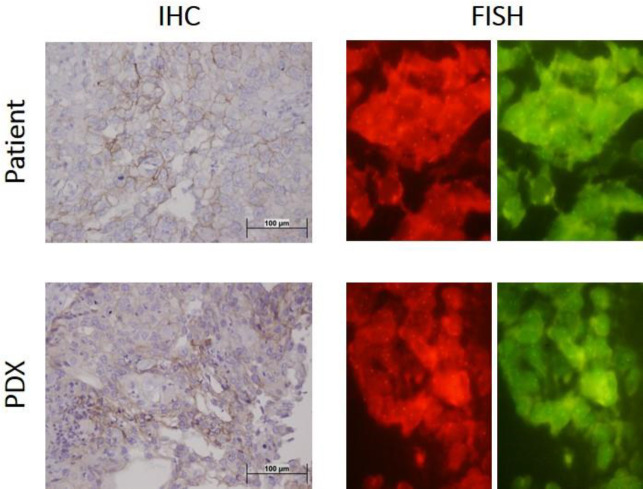
Representative images of HER2 expression, and gene copy number in a patient tumor and its corresponding patient-derived xenograft (PDX), obtained by immunohistochemistry (IHC) and fluorescence *in situ* hybridization (FISH) techniques. The low level of HER2 expression was found both in the patient sample and corresponding PDX (IHC score=1). The ratios of HER2 probe signal (red) to CEP17 signal (green) represent the amplification status of the HER2 gene (HER2/CEP17 ratio <2)

A low level of HER2 expression was found in human tumors and their corresponding PDXs, as assessed by IHC and FISH techniques. As shown in Figure 1, IHC score=1 showed the low level of HER2 protein expression in our PDX models, and also HER2/CEP17 ratio <2 indicated no amplification in gene copy number. Representative images of HER2 expression in different generations of PDXs developed from two patients are shown in Supplementary Figure 1. Also, detailed data regarding the expression of HER2 and some other markers in our PDX models were recently published (8). Notably, the antitumor activity of T-DM1, especially in the combination groups, was observed in our models with low level of HER2 expression. 

***Assessment of treatment efficacy *** 

**Figure 2:**
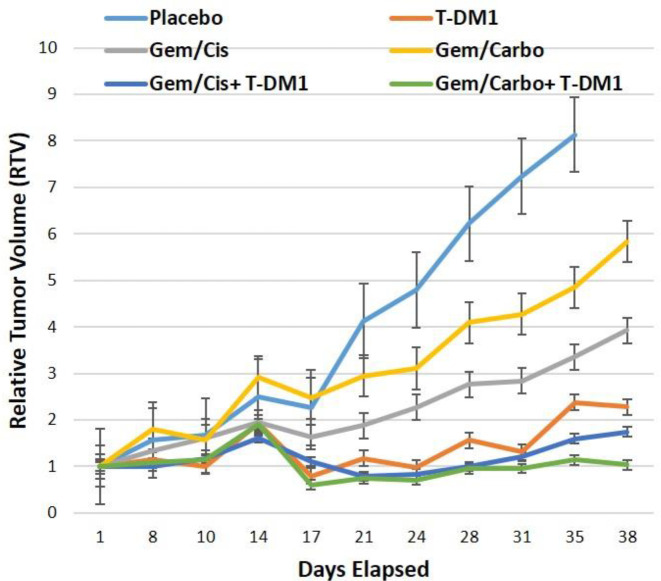
Mean of the relative tumor volume (RTV)±SEM for chemotherapy or T-DM1 alone and their combination groups, representing the tumor growth over time. RTV was defined as the mean tumor volume ratio between a given day and the day of treatment initiation. Cisplatin, carboplatin, gemcitabine, and T-DM1 were administered as 3 mg/kg, 10 mg/kg, 40 mg/kg, and 44 mg/kg, respectively

The treatment efficacy of chemotherapy or T-DM1 alone and their combination was evaluated on tumor growth *in vivo*. Tumor growth over time is indicated by plotting the mean of RTV±SEM per group (Figure 2). The mean RTVs for PDXs developed from two patients are separately shown in Supplementary Figure 2.

***Tumor growth inhibition*** 

**Figure 3:**
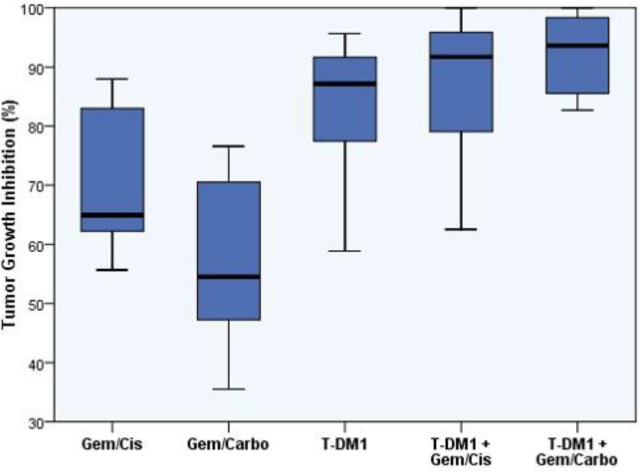
Box plot of tumor growth inhibition (TGI) for chemotherapy or T-DM1 alone, and their combination groups, representing the treatment efficacy at the end of the study. %TGI= 100 − (RTV treated/RTV control) *100. Cisplatin, carboplatin, gemcitabine, and T-DM1 were administered as 3 mg/kg, 10 mg/kg, 40 mg/kg, and 44 mg/kg, respectively

At study completion, TGI was calculated as a standard endpoint for therapeutic efficacy in PDXs. A box plot of TGI for all treatment groups is depicted in Figure 3. As indicated in Figure 3, monotherapy of T-DM1 induced a moderate antitumor activity in our PDX models with low level of HER2 expression (TGI_T-DM1 _= 82.9%; 95% confidence interval [CI]: 68.9-97.0). Although chemotherapy regimens induced a slight TGI when administered alone (TGI_Gem/Cis _= 70.7%; 95%CI: 53.4-88.1, and TGI_Gem/Carbo _= 56.4%; 95%CI: 40.6-72.2), interestingly, both regimens showed a strong antitumor activity when administered in combination with T-DM1 (TGI_Gem/Cis+T-DM1_ = 86.9%; 95% CI: 76.4-97.5, and TGI_Gem/Carbo+T-DM1_ = 92.3%; 95% CI: 85.0-99.5). Statistically significant differences were merely observed between the combination groups with Gem/Carbo regimen (*P*=0.019 for Gem/Cis+T-DM1, and *P*=0.006 for Gem/Carbo+T-DM1).  

***Tumor response*** 

Response status for each group is indicated in Table 1. As shown in the table, no response was observed in two chemotherapy groups. Notably, the observed OR rates were higher when T-DM1 was combined with Gem/Cis or Gem/Carbo regimens (62.5% for Gem/Cis+T-DM1, and 66.7% for Gem/Carbo+T-DM1) than when T-DM1 was used alone (16.7%). When compared with the placebo group, the OR rates of combination groups were statistically significant (*P*=0.026 for Gem/Cis+T-DM1, and *P*=0.021 for Gem/Carbo+T-DM1). So, no significant difference was observed between the placebo group with T-DM1 monotherapy with regard to the OR rate (*P*=0.462). 

***Assessment of treatment toxicity *** 

**Figure 4:**
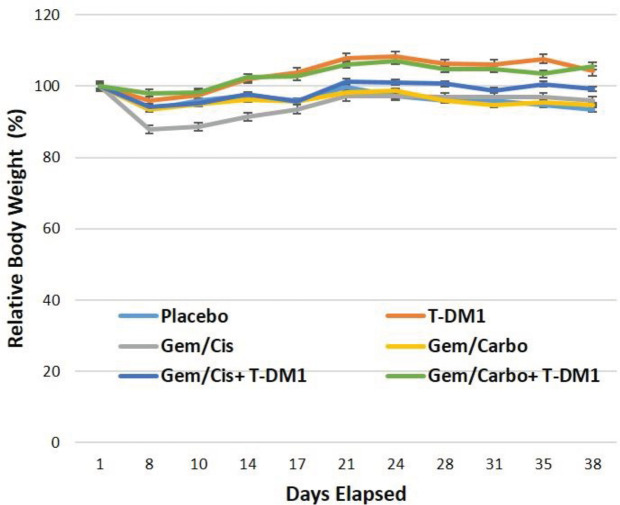
Mean of relative body weight (RBW) ±SEM for chemotherapy or T-DM1 alone and their combination groups, representing the treatment toxicity over time. RBW (%) = BWn/BW0*100. Cisplatin, carboplatin, gemcitabine, and T-DM1 were administered as 3 mg/kg, 10 mg/kg, 40 mg/kg, and 44 mg/kg, respectively

To assess the safety of different drugs used in this study, the mean of RBW±SEM was calculated for each group (Figure 4). The administered dose of each drug used alone or in combination groups was well tolerated in mice (3 mg/kg for cisplatin, 10 mg/kg for carboplatin, 40 mg/kg for gemcitabine, and 44 mg/kg for T-DM1). As shown in Figure 4, no treatment group induced a mean weight loss >20% of the initial weight. The mean of RBWs for PDXs developed from two patients are separately exhibited in Supplementary Figure 3.

**Table 1:** Response status for all study groups

**Group**	**PD (%)**	**SD (%)**	**PR (%)**	**CR (%)**	**OR (%)**	***P-*** **value** ^*^
Placebo	100	0	0	0	0	-
Gem/Cis	100	0	0	0	0	-
Gem/Carbo	100	0	0	0	0	-
T-DM1	66.6	16.7	16.7	0	16.7	0.462
T-DM1 + Gem/Cis	37.5	0	50	12.5	62.5	0.026
T-DM1 + Gem/Carbo	33.3	0	50	16.7	66.7	0.021

*Significance of OR rate between the placebo group with treatment groups was analyzed using Fisher’s exact test

PD: progressive disease; SD: stable disease; PR: partial response; CR: complete response; OR: overall response; Gem/Cis: Gemcitabine/Cisplatin; Gem/Carbo: Gemcitabine/Carboplatin

## Discussion

Some previous studies have demonstrated an important therapeutic potential for combination of an ADC and cytotoxic chemotherapy (17-20). The combination of chemotherapeutic agents with a drug targeting different molecular targets may increase tumor cell killing, reduce the likelihood of drug resistance and minimize overlapping toxicity (21). Moreover, previous studies have indicated that cytotoxic chemotherapy drugs can increase tumor expression of ADC-catabolizing proteases (7, 22). Consequently, it is likely that in order to obtain considerable effect in MIBC patients, platinum-based chemotherapy may need to be combined with a proper ADC. In a preclinical study, Hayashi *et al*. have suggested that . T-DM1 could be considered an attractive therapeutic target for bladder cancer (23). The most interesting finding in their study was that the HER2 expression was higher in cell lines with acquired cisplatin resistance cell line. Also, tumor growth of cisplatin-resistant cells was significantly inhibited by TDM1 in an orthotopic bladder cancer xenograft model. 

To the best of our knowledge, there is no preclinical study investigating the combined effect of T-DM1 and platinum-based chemotherapy. In previous similar studies, T-DM1 in combination with other agents demonstrated an enhanced antitumor activity in different tumor xenografts (24, 25). Phillips *et al*. showed the improved benefit of T-DM1 in combination with pertuzumab in xenograft models of breast cancer (24). In their study, dual targeting of HER2 with the combination of T-DM1 and pertuzumab resulted in synergistic inhibition of cell proliferation and induction of apoptotic cell death. Also, in another preclinical study, the combination of T-DM1 with PI3K/mTOR inhibitors enhanced its anti-tumor activity in breast cancer lines *in vitro* and as xenografts *in vivo* (25). DM1 is a potent inhibitor of microtubule assembly thereby causing cell death after degradation in the lysosomes (26, 27).

The combination therapy of gemcitabine, cisplatin, or carboplatin with an ADC was also tested *in vivo* in different xenograft models (17-20).  In two recent studies, the combination of gemcitabine with an anti-c-Met ADC (18), and anti-CD25 ADC (19) resulted in synergistic anti-tumor activity both *in vitro* and *in vivo*. Also, Rather *et al*. tested an ADC (M69-MMAE) alone and in combination with cisplatin in breast cancer xenograft. Although the ADC alone had a minimal anti-tumor effect, consistent with our study, it markedly enhanced the efficacy of cisplatin without toxicity (17). Moreover, in a SKOV3 xenograft model, the anti-tumor efficacy of platinum-based chemotherapy in combination with an anti-54F ADC (A1mcMMAF) was evaluated by Wan *et al*. (20). The authors indicated delayed progression and prolonged survival when anti-54F ADC was used in combination with carboplatin.  

In agreement with clinical studies comparing Gem/Carbo and Gem/Cis regimens in bladder cancer (28), Gem/Carbo induced less anti-tumor activity in our study (TGI_Gem/Carbo _= 56.4%; 95%CI: 40.6-72.2 vs. TGI_Gem/Cis _= 70.7%; 95%CI: 53.4-88.1); however, this difference was not statistically significant. Interestingly, Gem/Carbo regimen indicated a strong antitumor activity when administered in combination with T-DM1 (TGI_Gem/Carbo+T-DM1_ = 92.3%; 95% CI: 85.0-99.5). Since Gem/Carbo regimen is related to less toxicity compared with Gem/Cis regimen in clinical practice, the replacement of cisplatin by carboplatin combination therapy may be considered after clinical confirmation. 

In terms of tumor response outcome, although no response was observed in the chemotherapy alone groups, surprisingly, these chemotherapy regimens had the potential to induce substantial tumor response when combined with T-DM1 (62.5% for Gem/Cis+T-DM1, and 66.7% for Gem/Carbo+T-DM1). Notably, the addition of T-DM1 to the chemotherapy regimens exhibited a desirable response whereas a low level of HER2 expression was observed in both PDX models used in our study. In line with this observation, T-DM1 had a clear inhibitory effect in gastric cancer cells with a low level of HER2 expression (29). However, non-cleavable ADCs, such as T-DM1, are best suited for cancers with high expression of the target antigen (30). Thus, it is expected that a higher target expression in the tumor could result in an excellent response to treatment. The potential of T-DM1, as an anti-HER2 targeted therapy, to improve the efficacy of platinum-based chemotherapy in MIBC PDXs models with a low level of HER2 expression offers an exciting opportunity in the treatment of bladder cancer with even low HER2 expression. 

As a critical point, it should be noted that the utilization of T-DM1 as a non-cleavable ADC could balance the increased toxicity related to drug combination since the main advantage of non-cleavable versus cleavable ADCs is their improved plasma stability that results in reduced off-target toxicity. Importantly, our data indicated that despite the lack of any dose reduction in the combination groups, no increased toxicity was observed compared with other groups. However, to resolve clinical safety considerations, a phase 1 dose-escalation trial should be performed.  

Since the intravenous injection route is technically challenging and can be stressful for animals, particularly for immunodeficient mice, the intraperitoneal injection route was used for drug administration. Moreover, intraperitoneal injection is more reproducible and easy to master compared with the intravenous route. In this light, previous studies concluded that intraperitoneal injection of drugs in experimental animals is a justifiable route for pharmacological and proof-of-concept studies where the goal is to evaluate the effect of target engagement rather than the properties of a drug formulation or its pharmacokinetics (31).

Some limitations should be considered in this study. Although our PDX models reserved mostly histopathological characteristics of patient tumors, we used PDX tissues originating from only two patients. Moreover, despite significant *P*-values, the fairly wide range of CI for TGIs should be considered. This wide range of CI may be related to the small sample size. However, our preclinical data could provide substantial new insight and rationale for clinical investigation. 

## Conclusion

Addition of TDM1 to platinum-containing regimens showed promising antitumor efficacy in preclinical models of MIBC. In this light, running well-designed clinical trials is warranted to prove the efficacy and safety of this combination in patients with bladder cancer. Also, our preclinical data may provide substantial new insight and rationale for combining ADCs and cytotoxic chemotherapy. 

## Authors’ Contributions

AR, SM, and AB designed the experiments; AR, SM, MP, and MZ performed experiments and collected data; AR, SM, BM and AB discussed the results, supervised, directed, and managed the study; AR, SM, MP, BM, MZ, and AB approved the final version of the manuscript to be published.

## Conflicts of Interest

The authors report no conflicts of interest.
